# Effect of Dietary Vitamin A, Breed, and Kidney Function on the Onset of Urinary Vitamin A Excretion and the Vitamin A Transport Complex in Growing Dogs

**DOI:** 10.1111/jpn.70093

**Published:** 2026-07-16

**Authors:** Jens Raila, Haydar Özpinar, Florian J. Schweigert

**Affiliations:** ^1^ Institute of Nutritional Science University of Potsdam Potsdam Germany; ^2^ Department of Nutrition and Dietetics Istanbul Aydin University Istanbul Turkey

**Keywords:** blood, dog, retinol‐binding protein 4, Tamm‐Horsfall protein, urine, vitamin A

## Abstract

Vitamin A (retinol and retinyl esters) is present in substantial amounts in the urine of healthy adult dogs, but the mechanisms affecting its excretion are poorly understood. To assess whether the onset of urinary vitamin A excretion is dependent on dietary vitamin A intake, breed, and/or parameters of kidney function, 48 puppies from two breeds, the Labrador Retriever and the Miniature Schnauzer were included. Dogs were given four diets with increasing vitamin A content (5000; 12,500; 75,000 and 100,000 IU vitamin A/1000 kcal metabolizable energy). Plasma and urine samples were collected and the onset of a continuous vitamin A excretion in urine was determined for each dog. Vitamin A concentration was determined by HPLC. Concentrations of creatinine, urea, protein and albumin were determined by colorimetric assays. Retinol‐binding protein 4 (RBP4) and Tamm‐Horsfall protein (THP) were measured by semiquantitative Western blot. Plasma vitamin A concentrations increased with increased dietary vitamin A intake (*p* < 0.001), but the onset of urinary vitamin A excretion (*p* = 0.158) and the amount of vitamin A excreted at this time point were not different between the feeding groups (*p* = 0.128) or breed (*p* = 0.279). Concentrations of creatinine, urea, protein and albumin in plasma and protein in urine were not different between groups. There were no differences in the concentrations of RBP4 in plasma (*p* = 0.309), RBP4 in urine (*p* = 0.345) and THP in urine (*p* = 0.384). The onset of vitamin A excretion in the urine of dogs seems to be not directly associated with the plasma vitamin A concentration and, thus not simply explained by dietary vitamin A intake alone. The vitamin A excretion is not breed specific and not dependent on kidney function or the occurrence of transport proteins in plasma (RBP4) or urine (THP). Thus, the onset and the amount of vitamin A excreted are determined by factors that remain to be elucidated.

## 1 Introduction

Vitamin A is an essential fat‐soluble micronutrient that is crucial for various life processes such as vision, cell growth, epithelia differentiation, embryonic development and immune cell maturation (Blomhoff and Blomhoff 2008). All higher animals are unable to synthesize vitamin A de novo and have to acquire vitamin A from the diet, either as preformed retinyl esters from animal sources or as provitamin A carotenoids synthesized in plants (von Lintig 2012). Because of its crucial role, the vitamin A concentration in the blood is homeostatically regulated by a high‐affinity carrier, retinol‐binding protein (RBP4), which guarantees a constant and continuous supply of retinol from storage sites (mainly the liver) to target tissues (D'Ambrosio et al. 2011). RBP4 has a molecular mass of about 21 kDa and belongs to the lipocalin family of proteins that transport small hydrophobic molecules in various biological fluids (Flower 1996). RBP4 interacts not only with retinol, but also forms a complex with transthyretin (TTR), which stabilizes the RBP4‐retinol‐complex (*holo*‐RBP4) to prevent its removal by glomerular filtration and subsequent excretion in the urine (Raila et al. 2005). The cellular uptake of retinol in peripheral tissues is mediated by a RBP4 receptor, which was identified as a multi‐transmembrane domain protein called STRA6 (Kawaguchi et al. 2007). STRA6 removes retinol from RBP4 and transports it across the plasma membrane, where retinol can be further metabolized into all‐*trans‐* and 9‐*cis*‐retinoic acid, which act as ligands for transcriptional factors (RARs and RXRs) in the regulation of target gene expression (Sun 2012).

In comparison with other mammals dogs exhibit a distinctive pattern of vitamin A transport during fasting, characterized by the presence of not only *holo*‐RBP4, but also substantial amounts of circulating retinyl esters (Schweigert 1988; Wilson et al. 1987). These lipoprotein‐bound retinyl esters make up > 70% of the total vitamin A concentration and are not homeostatically regulated as *holo*‐RBP4, but are dependent on the actual dietary vitamin A intake (Morris et al. 2012; Schweigert and Bok 2000). In humans and rodents, comparably high plasma retinyl ester concentrations are mainly observed during postprandial lipemia or under conditions of chronic vitamin A intoxication (Krasinski et al. 1990; Mallia et al. 1975; Smith and Goodman 1976). The apparent tolerance of dogs to comparatively high circulating retinyl ester concentrations raises questions about the mechanisms involved in maintaining vitamin A homeostasis in this species (Cline et al. 1997; Morris et al. 2012; Schweigert 1988). However, this feature should not be generalized to all carnivore species, because vitamin A handling and susceptibility to hypervitaminosis A differ markedly between species. Notably, chronic vitamin A intoxication is well documented in cats resulting in the formation of diffuse exostosis on the cervical vertebrae, periosteal reactions of the long bones and signs of liver fibrosis (Seawright et al. 1967; Pobisch and Onderscheka 1976; Corbee et al. 2014). In this context, the urinary elimination of vitamin A in the form of retinol and retinyl esters has been proposed as one possible mechanism contributing to vitamin A handling in dogs (Schweigert et al. 1991).

Retinol and retinyl esters are fat‐soluble compounds that have been shown to be associated with Tamm‐Horsfall protein (THP) in the urine of dogs. THP is a membrane protein that is synthesized by cells in the distal renal tubules and is released in the urine (Schweigert et al. 2002). However, two feeding studies investigated whether the level of vitamin A in plasma affects the vitamin A excretion in urine, but no association between the concentrations of vitamin A in plasma and the amount of vitamin A excreted in urine was found (Raila et al. 2002; Schweigert and Bok 2000). Importantly, urinary elimination is unlikely to represent the primary route of vitamin A regulation, because vitamin A homeostasis is mainly governed by hepatic storage of retinyl esters, regulated release of retinol bound to RBP4, tissue uptake, and enzymatic metabolism of retinoids (D'Ambrosio et al. 2011). Therefore, urinary vitamin A excretion in dogs should be interpreted as an unusual physiological phenomenon rather than assumed to be the dominant detoxification pathway.

More recently, a long‐term feeding trial assessed the safety of dietary vitamin A in growing dogs and reported a high individual variability in the excretion of vitamin A, even though dogs were supplemented with equal amounts of vitamin A in the diet (Morris et al. 2012). The cause of this observation is unclear, and it was assumed that this might be due to differences in the expression and/or metabolism of the proteins involved in the transport of vitamin A in dogs.

In the present study, we therefore determined the individual age at the onset of urinary vitamin A excretion in puppies by reanalyzing the previously reported data (Morris et al. 2012) and measured the concentrations of RBP4 in plasma as well as RBP4 and THP in urine at this time point. We hypothesized that puppies exposed to higher dietary vitamin A would show an earlier onset of urinary vitamin A excretion and that this onset might additionally vary with breed or selected indicators of renal function.

## 2 Materials and Methods

### 2.1 Animals and Study Design

The present study is an extensive secondary analysis of existing data available from a previous study that described the safety evaluation of vitamin A in growing dogs up to 52 weeks of age (Morris et al. 2012). The study included 48 puppies from two breeds namely, Miniature Schnauzers (MS) and Labrador Retrievers (LR). The population demographics and research protocol were described in detail elsewhere (Morris et al. 2012) and are presented in Table 1. Briefly, all dogs were housed with their mothers until 8 weeks of age and afterwards in pairs in environmentally enriched kennels with free access to outdoor areas. After weaning, all puppies received a balanced diet (Perfect Fit Junior; Mars GmbH) and were randomized within litter to one of the following feeding groups, namely group A: 5000 IU vitamin A per 1000 kcal metabolizable energy (ME); group B: 12,500 IU vitamin A per 1000 kcal ME; group C: 75,000 IU vitamin A per 1000 kcal ME, and group D 100,000 IU vitamin A per 1000 kcal ME. Free access to drinking water was given at all times. The puppies underwent physical examination before the start of the trial and every 4 weeks thereafter. No signs of disease were revealed by physical and complete biochemical and hematological examination or urinalysis up to 52 weeks of age. For the present secondary analysis, the onset of urinary vitamin A excretion was defined as the first sampling time point at which vitamin A was continuously detectable in urine in the longitudinal series of an individual dog. The research protocol was approved by the WALTHAM® Internal Ethics Committee.

**Table 1:** Population demographics of the dogs completing the study.

	Group A	Group B	Group C	Group D
n	15	11	11	11
Breed				
MS	8	6	6	5
LR	7	5	5	6
Sex				
MS	8	5	6	6
LR	7	6	5	5
Dietary vitamin A (IU)^a^	5000	12,500	75,000	100,000

Abbreviations: LR Labrador retriever; MS Miniature schnauzer.

^a^ Calculated on the basis of 1000 kcal metabolizable energy

### 2.2 Blood and Urine Collection

Blood was sampled from a jugular vein after an overnight fast at week 8 (before dietary vitamin A supplementation) and at weeks 10, 12, 14, 16, 20, 26, 35, and 52. For biochemical analysis, blood samples were collected into tubes containing lithium heparin. Plasma was prepared by centrifuging the samples at 2000 × *g* for 10 min at 4°C and was immediately used for analysis. For vitamin A determination, blood was sampled into light‐proof tubes containing no anticoagulant. Serum was prepared by centrifuging the samples at 2000 × *g* for 10 min at room temperature and stored at −80°C until analysis within 4 weeks. Urine was collected 1 day before blood sampling, following a 16 h fast, using a free‐catch method. The samples were placed in light‐proof tubes and stored at −80°C until analysis within 4 weeks.

### 2.3 Plasma Biochemical Analysis and Urinalysis

Plasma creatinine, urea, total protein and albumin concentrations were measured by the use of an automated analysis system (Olympus AU400; Olympus Inc., Tokyo, Japan). The concentrations of urinary protein were assessed via Bradford colorimetric assay (Bradford Protein‐Assay, Bio‐Rad, Munich, Germany) and urinary creatinine was determined by the standard Jaffé reaction (Fisher Diagnostics, Middletown, USA).

### 2.4 Determination of Retinol and Retinyl Esters

Vitamin A (retinol and retinyl esters) in plasma and urine was determined using a modified gradient reversed‐phase HPLC‐system (Waters, Eschborn, Germany) after organic extraction as previously described (Raila et al. 2005). Briefly, retinol and retinyl esters were extracted twice with n‐hexane from plasma and urine and separated on a C30 column (5 µm, 250 × 4.6 mm, YMC, Wilmington, USA). The solvent system consisted of solvent A with methanol/water (90:10, v/v, 0.4 g/l ammonium acetate in H_2_O) and solvent B with methanol/methyl‐*tert*‐butyl‐ether/water (8:90:2, v/v/v, 0.1 g/l ammonium acetate in H_2_O) at a column flow rate of 0.2 ml/min. Retinol and retinyl esters were quantified by measuring the absorption at 325 nm by a photodiode array detector (Model 996, Waters) using external standards (Sigma, Deisenhofen, Germany). Results were compared to standard reference material (SMR 968a fat‐soluble vitamins in human serum; National Institute of Standards and Technology, Gaithersburg, USA). The detection limits for retinol was 4.0 ng/ml and for retinyl palmitate 6.8 ng/ml. The recovery rate was above 95% for all components. All solvents or chemicals used for extraction or HPLC were of high‐purity commercial grade (Roth, Karlsruhe, Germany).

### 2.5 Determination of Retinol‐Binding Protein (RBP4)

To assess the presence of RBP4 in plasma and urine, 12% sodium dodecyl sulfate polyacrylamide gel electrophoresis (SDS‐PAGE) separation was performed. After SDS‐PAGE, the separated proteins were electroblotted onto a polyvinylidene difluoride (PVDF) membrane and Tris‐buffered saline (TBS) containing 0.1% Tween 20 (TBST) and 5% defatted milk was used to block nonspecific binding sites on the blot. The membrane was then incubated with a peroxidase‐coupled rabbit anti‐human RBP4 IgG (Biozol, Eching, Germany; 1:300 diluted in 0.1% TBST) for 1 h at room temperature. Antibody binding was visualized using the Luminol reaction (BM Chemiluminescence Blotting Substrate, Roche Diagnostics, Mannheim, Germany). Band intensity of RBP4 was read with an imager (Bio‐Rad, Munich, Germany) and analyzed with the Bio‐Rad ChemiDoc XRS software (Raila et al. 2005).

### 2.6 Determination of Tamm–Horsfall Protein (THP)

For determination of the THP concentrations, the urine proteins were separated by SDS‐PAGE, transferred onto a PVDF membrane and blocked with 5% nonfat milk in 0.1% TBST. The membrane was then incubated with primary sheep anti‐THP antibodies (Chemicon, Temecula, USA) at 4°C overnight. After being washed in 0.1% TBST, the blots were incubated with peroxidase‐conjugated rabbit‐anti‐sheep antibodies (DakoCytomatio, Hamburg, Germany) for 1 h at room temperature. After final wash with 0.1% TBST, antibody binding was visualized with the BM Chemiluminescence Blotting Substrate (Roche Diagnostics). The band intensity of THP was read with an imager (Bio‐Rad) and analyzed with the Bio‐Rad ChemiDoc XRS software 1.1. Specificity of the immunoblot was determined by co‐migration with purified human THP (Chemicon) as previously described (Raila et al. 2014). The intra‐ and inter‐assay precisions had a coefficient of variation of 7.4% and 15.7%, respectively, for THP standard concentrations of 20 ng/µl.

### 2.7 Statistical Analyses

Statistical analyses were performed by SPSS Statistics, version 19.0 (Munich, Germany). The Kolmogorov‐Smirnov test was used to assess whether the data were normally distributed or not normally distributed. All serum values as well as age, body weight, vitamin A intake and UP/UC were normally distributed and differences between the groups were analyzed using the one‐way ANOVA with Scheffé post hoc correction. Urine values for retinol, retinyl ester, total vitamin A, RBP4 and THP were not normally distributed. Therefore, the nonparametric Kruskal‐Wallis followed by the Mann‐Whitney *U*‐test was used to describe differences between the groups. All urine values were normalized for urinary creatinine (UC) excretion. Differences were considered significant at a *p* value of ≤ 0.05.

## 3 Results

### 3.1 Age, Body Weight and Dietary Vitamin A Intake at Onset of Urinary Vitamin A Excretion

The beginning of urinary vitamin A excretion was irrespective of feeding group and was between the 16th and 26th week age. The average age at onset of vitamin A excretion was 19.8 ± 3.63 (mean ± SD) weeks of age. Due to the high individual variability, the age of onset of urinary vitamin A excretion did not differ significantly between feeding groups (*p* = 0.158) or breeds (*p* = 0.279; Table 2). There were no significant differences in body weight within breed between the feeding groups, MS (*p* = 0.539) and LR (*p* = 0.085), but, as anticipated, there was a significant effect of breed, with LR having higher body weights than MS (*p* < 0.001). As a result of the feeding regime, the vitamin A intake was different among the groups, with values that were lowest in group A and highest in group D (*p* < 0.001; Table 2).

**Table 2:** Age, body weight and Vitamin A intake at onset of urinary Vitamin A excretion in miniature schnauzer (MS) and labrador retriever (LR) puppy dogs.

	Group A	Group B	Group C	Group D	*p‐*value
Age (weeks)	21.2 ± 3.84	20.0 ± 3.46	19.4 ± 2.84	18.3 ± 3.20	0.217
Body weight					
MS (kg)	5.90 ± 1.29	5.38 ± 1.25	5.19 ± 1.44	4.80 ± 1.43	0.539
LR (kg)	19.0 ± 4.00	14.8 ± 2.22	15.2 ± 0.76	14.6 ± 4.31	0.085
Vitamin A intake (IU/kg BW)	609 ± 96.5^a^	1.510 ± 296^b^	9.988 ± 1.661^c^	13.976 ± 2.205^d^	< 0.001

*Note:* Values are means ± standard deviations. Means in a row without a common superscript (a–d) differ significantly at *p* < 0.001, by one‐way ANOVA with post hoc test.

Abbreviations: LR, Labrador retriever; MS, Miniature schnauzer.

### 3.2 Parameters of Kidney Function in Plasma and Urine

At the onset of urinary vitamin A excretion, there was no significant effect of feeding group on the concentrations of creatinine (*p* = 0.547), urea (*p* = 0.133), total protein (*p* = 0.673) and albumin (*p* = 0.399) in plasma of dogs (Table 3). In urine, the UP/UC levels were also not different between groups (Table 3).

**Table 3:** Biochemical parameters of kidney function in plasma and urine of dogs.

	Group A	Group B	Group C	Group D	*p* value
Creatinine (µmol/l)	63.1 ± 9.89	59.7 ± 6.23	57.5 ± 7.02	54.0 ± 8.00	0.547
Urea (mmol/l)	4.55 ± 0.74	4.09 ± 0.50	4.15 ± 0.89	3.91 ± 0.60	0.133
Total protein (g/l)	53.8 ± 3.41	53.5 ± 3.42	52.8 ± 3.07	52.3 ± 2.64	0.673
Plasma albumin (g/l)	28.2 ± 1.48	27.9 ± 1.79	27.5 ± 1.97	27.1 ± 1.28	0.399
UP/UC	0.14 ± 0.06	0.16 ± 0.05	0.16 ± 0.10	0.19 ± 0.06	0.377

*Note:* Values are means ± standard deviations. There were no significant differences in any parameter between the groups by one‐way ANOVA. UP/UC, ratio between urinary protein to urinary creatinine.

### 3.3 Vitamin A in Serum and Urine

The dietary supplementation with different amounts of vitamin A (5000 to 100,000 IU/1000 kcal ME) resulted in group‐specific differences in serum retinyl ester (*p* < 0.001) and total vitamin A concentrations (*p* < 0.001). By contrast, serum retinol was not affected by dietary vitamin A intake (*p* = 0.876; Table 4). The mean concentrations of vitamin A in urine, depicted as urinary vitamin A to urinary creatinine ratio, tended to be higher with increasing amounts of vitamin A intake, but the effect did not reach statistical significance (*p* = 0.128; Table 5). Moreover, there were no significant differences in the urinary retinol‐to‐urinary creatinine (*p* = 0.384) and urinary retinyl ester‐to‐urinary creatinine ratios (*p* = 0.829; Table 5).

**Table 4:** Concentrations of total Vitamin A, retinol, retinyl esters, and retinol‐binding protein (RBP4) in serum.

	Group A	Group B	Group C	Group D	*p‐*value
Total vitamin A (µg/ml)	4.42 ± 1.33^a^	7.80 ± 1.62^b^	13.1 ± 3.77^c^	20.8 ± 5.96^d^	< 0.001
Retinol (µg/ml)	0.94 ± 0.27	0.89 ± 0.30	0.97 ± 0.33	0.89 ± 0.24	0.876
Retinyl ester (µg/ml)	3.48 ± 1.23^a^	6.91 ± 1.69^b^	12.1 ± 3.74^c^	19.9 ± 5.99^d^	< 0.001
RBP4 (µg/ml)	42.2 ± 8.84	40.9 ± 7.38	43.9 ± 11.3	47.7 ± 7.98	0.246

*Note:* Values are means ± standard deviations. Means in a row without a common superscript (a–d) differ significantly at *p* < 0.001, by one‐way ANOVA and *post hoc* test.

Abbreviations: RBP4, retinol‐binding protein.

**Table 5:** Concentrations of Vitamin A, retinol, retinyl ester, RBP4 and THP in urine of puppy dogs.

	Group A	Group B	Group C	Group D	*p‐*value
Vitamin A/UC (µg/mg)	0.015 (0.008–0.121)	0.017 (0.005–0.106)	0.026 (0.006–0.161)	0.039 (0.006–0.136)	0.128
Retinol/UC (µg/mg)	0.009 (0.0–0.028)	0.005 (0.0−0.062)	0.017 (0.0–0.051)	0.016 (0.0–0.096)	0.384
Retinyl ester/UC (µg/mg)	0.008 (0.0–0.121)	0.006 (0.0–0.062)	0.025 (0.0–0.109)	0.022 (0.0–0.136)	0.829
URBP4/UC (µg/mg)	0.0 (0.0)	0.0 (0.0)	0.0 (0.0)	0.0 (0.0)	0.345
THP/UC (µg/mg)	17.5 (2.01–88.9)	22.8 (9.93–77.6)	18.1 (6.77–26.5)	15.9 (7.98–77.6)	0.760

*Note:* Values are means (25th−75th percentile). There were no significant differences in any parameter between the groups by the nonparametric Kruskal‐Wallis test.

Abbreviations: UC, urinary creatinine, URBP4, urinary retinol‐binding protein; THP, Tamm‐Horsfall protein.

### 3.4 RBP4 and THP Concentrations in Serum and Urine

Serum RBP4 was not different between the feeding groups (*p* = 0.309; Table 4) and there were no differences in the concentrations of serum RBP4 between MS and LR (*p* = 0.188). The excretion of RBP4 in the urine, as depicted as urinary RBP4‐to‐urinary creatinine (URBP4/UC) ratio, was negligible and not different among the groups (*p* = 0.345). Urinary THP, depicted as THP/UC ratio, was present in substantial amounts, but was not different among the feeding groups (*p* = 0.384, Table 5).

## 4 Discussion

The urinary excretion of vitamin A in the form of retinol and retinyl esters is a well‐known physiological phenomenon frequently observed in healthy adult dogs and some other canine species such as foxes and raccoon dogs (Raila et al. 2000; Schweigert et al. 1991). In humans, vitamin A is only excreted with the urine as metabolized retinol compounds or has been observed in patients suffering from severe infections during febrile proteinuria (Lambert and De Leenheer 1985; Stephensen et al. 1994). The biological relevance and the mechanisms regulating the renal retinol and retinyl ester excretion in dogs are still unclear. Vitamin A homeostasis is primarily maintained through hepatic storage of retinyl esters, regulated release of retinol bound to RBP4, tissue uptake, and enzymatic metabolism of bioactive retinoids (D'Ambrosio et al. 2011). Therefore, urinary elimination of retinol and retinyl esters in dogs should be interpreted as an unusual species‐associated phenomenon rather than assumed to be the principal route protecting against vitamin A excess. Against this physiological background, the present study was designed to test whether the onset of this unusual urinary excretion pattern is associated with dietary vitamin A intake, breed, or selected indicators of renal function. The results clearly show that increasing levels of vitamin A in the diet were reflected by increasing concentrations of vitamin A in serum, with the highest in group D and the lowest in group A. This observation is mainly due to an effect of vitamin A intake on serum retinyl esters, but not serum retinol, which is in agreement with our previous investigations (Morris et al. 2012; Raila et al. 2002; Schweigert and Bok 2000). Based on this result, we hypothesized that growing dogs receiving a diet high in vitamin A (e.g., group D) would excrete vitamin A in urine earlier than those receiving a diet low in vitamin A (e.g., group A). Contrary to our expectations, the age at the onset of urinary vitamin A excretion did not differ significantly between the groups, indicating that the beginning of urinary vitamin A excretion in puppies is not related to plasma vitamin A and thus dietary vitamin A intake. This is an important finding of the study and has not been reported previously. However, it should be noted that the age at the first detection of urinary vitamin A was highly variable, mainly due to the relatively long time period between urine sampling (weeks 16, 20 and 26), which limits the possibility of narrowing the start of vitamin A excretion in individual dogs.

The present study further shows that breed does not influence the first urinary vitamin A excretion, although at this time point, the body weight differed remarkably between MS and LR. In general, the body weight is correlated with both, kidney weight and the absolute number of glomeruli in dogs, but the renal blood flow (RBF) and thus glomerular filtration rate (GFR) per nephron is constant regardless of the size of the mammal (Holt and Rhode 1976; Horster and Valtin 1971). Therefore, breed had also no effect on the biochemical parameters of kidney function, such as plasma creatinine and plasma urea. Taken together, these findings argue against a simple breed‐ or size‐dependent explanation for the onset of urinary vitamin A excretion.

The results further show that the concentrations of vitamin A excreted were not significantly different between the feeding groups, indicating that the amount of vitamin A excreted is likewise not directly explained by serum vitamin A concentrations or dietary vitamin A intake alone. This agrees with our previous investigations showing that urinary vitamin A excretion does not cease if dogs are fed a diet below the NRC (2006) requirements of daily 75 IU vitamin A/kg BW (Schweigert and Bok 2000) and that supplementation with a single oral dose of 3000 IU vitamin A/kg BW is not sufficient to provoke vitamin A excretion in urine (Raila et al. 2002). Despite comparable plasma vitamin A concentrations, the amount of vitamin A excreted varied substantially among dogs, indicating that factors other than the dietary vitamin A supply may contribute to this condition. One possible explanation is that urinary vitamin A excretion reflects inter‐individual differences in tissue handling of vitamin A rather than direct exposure alone. In this context, both hepatic buffering and renal vitamin A storage capacity may be relevant. The concentrations of vitamin A in canine kidney tissue have been shown to increase continuously during growth, reaching adult values only later in development (Schweigert et al. 1998). Because no tissue samples were available in the present study, it remains speculative whether hepatic storage, renal storage, altered retinoid metabolism, or a combination of these factors contributes to the timing and magnitude of urinary vitamin A excretion. Little is known about the molecular basis mediating the renal vitamin A excretion in dogs. Because of the differences in the pattern of retinyl esters in plasma (predominantly retinyl stearate) compared to urine (predominantly retinyl palmitate), it seems to be a highly selective process. Together with the absence of substantial amounts of RBP4 in urine, this finding indicates that vitamin A excretion in dogs is not merely a simple filtration process at the renal glomerulus. The finding that retinol and retinyl esters in urine are associated with THP points to a more specific mechanism of renal vitamin A elimination (Schweigert et al. 2002).

Immunohistochemical studies of dog kidney sections have shown that THP is mainly synthesized in the thick ascending limb of loop in distal tubules indicating that tubular secretion is the renal disposal of urinary vitamin A (Raila et al. 2003). In the present study, we confirmed that THP is physiologically excreted in canine urine (Schweigert et al. 2002). As observed for urinary vitamin A, urinary THP/UC also showed considerable inter‐individual variation and did not differ significantly among feeding groups. However, the measured values were comparable to those reported previously in healthy adult dogs (Raila et al. 2014). This suggests that renal maturation is already largely complete at the time urinary vitamin A excretion begins. This interpretation is consistent with earlier observations that renal blood flow, glomerular filtration rate, and para‐aminohippurate clearance in dogs approach mature values at approximately 12–16 weeks after birth (Horster and Valtin 1971; Jose et al. 1971). Moreover, urinary THP was already detectable at week 8 of age onwards (data not shown), indicating that the carrier protein is present in urine before the mechanisms of vitamin A begin. It is therefore speculative whether THP is present in urine as *holo*‐ (with vitamin A) and *apo*‐ (without vitamin A) variant, but this should be investigated in further studies.

In the present study, the concentrations of RBP4 in serum were not significantly different between the feeding groups. This can be explained by the fact that RBP4 is the only specific transport protein for retinol in the serum (D'Ambrosio et al. 2011) and that the release of *holo*‐RBP4 from the liver is tightly regulated despite marked variation in dietary vitamin A supply or hepatic reserve stores (O'Byrne and Blaner 2013). Therefore, as noted above, the dietary intervention with different vitamin A levels affected serum retinyl ester, but not retinol. Moreover, the circulating *holo*‐RBP4 did not appear to contribute to urinary retinol excretion, because RBP4 was not detected in the urine samples. This further indicates that urinary retinol is not bound to RBP4 and that the glomerular filtration of *holo*‐RBP4 is unlikely to represent the mechanism by which retinol is excreted in urine. The occurrence of RBP4 in urine has mainly been described in dogs with chronic renal disease (Raila et al. 2003; Smets et al. 2010). In this setting, the fractional excretion of RBP4 is increased because of impaired reabsorption in the proximal tubules (Raila et al. 2010). Due to this mechanism, urinary RBP4 has been proposed as a marker of kidney damage (De Loor et al. 2013), although its role in the diagnosis of canine nephropathy remains controversial.

Taken together, the results clearly show that increasing amounts of dietary vitamin A resulted in increasing serum concentrations of retinyl esters and, thus total vitamin A in puppies. However, serum vitamin A concentration was not related to either the age at the onset of urinary vitamin A excretion or the amount of vitamin A excreted at this time point. These findings do not support the concept that the onset of urinary vitamin A excretion in growing dogs is driven simply by dietary vitamin A intake. Based on the kidney function data, together with the observed handling of RBP4 and THP, the emergence of urinary vitamin A excretion appears more likely to reflect a developmental feature of renal vitamin A handling than a direct response to circulating vitamin A concentrations alone. Further studies are required to investigate the molecular mechanisms underlying this physiological phenomenon, especially the processes regulating the transfer of retinol and retinyl esters across the blood–urine barrier and the possible contribution of hepatic and renal vitamin A storage.

## Ethics Statement

The research protocol was evaluated and approved by the WALTHAM® Internal Ethics Committee.

## Data Availability

Data are unavailable due to privacy or ethical restrictions.

## References

[jpn70093-bib-0001] Blomhoff, R. , and H. K. Blomhoff . 2006. “Overview of Retinoid Metabolism and Function.” Journal of Neurobiology 66, no. 7: 606–630. 10.1002/neu.20242. 16688755

[jpn70093-bib-0002] Cline, J. L. , G. L. Czarnecki‐Maulden , J. M. Losonsky , C. R. Sipe , and R. A. Easter . 1997. “Effect of Increasing Dietary Vitamin A on Bone Density in Adult Dogs.” Journal of Animal Science 75, no. 11: 2980–2985. 10.2527/1997.75112980x. 9374313

[jpn70093-bib-0003] Corbee, R. J. , M. A. Tryfonidou , G. C. M. Grinwis , et al. 2014. “Skeletal and Hepatic Changes Induced by Chronic Vitamin A Supplementation in Cats.” Veterinary Journal 202, no. 3: 503–509. 10.1016/j.tvjl.2014.09.029. 25457260

[jpn70093-bib-0004] D'Ambrosio, D. N. , R. D. Clugston , and W. S. Blaner . 2011. “Vitamin A Metabolism: An Update.” Nutrients 3, no. 1: 63–103. 10.3390/nu3010063. 21350678 PMC3042718

[jpn70093-bib-0005] Flower, D. R. 1996. “The Lipocalin Protein Family: Structure and Function.” Biochemical Journal 318, no. Pt 1: 1–14. 10.1042/bj3180001. 8761444 PMC1217580

[jpn70093-bib-0006] Holt, J. P. , and E. A. Rhode . 1976. “Similarity of Renal Glomerular Hemodynamics in Mammals.” American Heart Journal 92, no. 4: 465–472. 10.1016/s0002-8703(76)80046-4. 961585

[jpn70093-bib-0007] Horster, M. , and H. Valtin . 1971. “Postnatal Development of Renal Function: Micropuncture and Clearance Studies in the Dog.” Journal of Clinical Investigation 50, no. 4: 779–795. 10.1172/jci106549. 5547275 PMC291992

[jpn70093-bib-0008] Jose, P. A. , A. G. Logan , L. M. Slotkoff , L. S. Lilienfield , P. L. Calcagno , and G. M. Eisner . 1971. “Intrarenal Blood Flow Distribution in Canine Puppies.” Pediatric Research 5, no. 8: 335–344. 10.1203/00006450-197108000-00001. 5146081

[jpn70093-bib-0009] Kawaguchi, R. , J. Yu , J. Honda , et al. 2007. “A Membrane Receptor for Retinol Binding Protein Mediates Cellular Uptake of Vitamin A.” Science 315, no. 5813: 820–825. 10.1126/science.1136244. 17255476

[jpn70093-bib-0010] Krasinski, S. D. , J. S. Cohn , E. J. Schaefer , and R. M. Russell . 1990. “Postprandial Plasma Retinyl Ester Response Is Greater in Older Subjects Compared With Younger Subjects. Evidence for Delayed Plasma Clearance of Intestinal Lipoproteins.” Journal of Clinical Investigation 85, no. 3: 883–892. 10.1172/jci114515. 2312731 PMC296506

[jpn70093-bib-0011] Lambert, W. E. , and A. P. De Leenheer . 1985. “Demonstration of Retinoic Acid Isomers in Human Urine Under Physiological Conditions.” Experientia 41, no. 3: 359–360. 10.1007/bf02004504. 3972081

[jpn70093-bib-0012] von Lintig, J. 2012. “Provitamin A Metabolism and Functions in Mammalian Biology.” American Journal of Clinical Nutrition 96, no. 5: 1234S–1244S. 10.3945/ajcn.112.034629. 23053549 PMC3471205

[jpn70093-bib-0013] De Loor, J. , S. Daminet , P. Smets , B. Maddens , and E. Meyer . 2013. “Urinary Biomarkers for Acute Kidney Injury in Dogs.” Journal of Veterinary Internal Medicine 27, no. 5: 998–1010. 10.1111/jvim.12155. 23952327

[jpn70093-bib-0014] Mallia, A. K. , J. E. Smith , and D. W. Goodman . 1975. “Metabolism of Retinol‐Binding Protein and Vitamin A During Hypervitaminosis A in the Rat.” Journal of Lipid Research 16, no. 3: 180–188. 1127357

[jpn70093-bib-0015] Morris, P. J. , C. Salt , J. Raila , et al. 2012. “Safety Evaluation of Vitamin A in Growing Dogs.” British Journal of Nutrition 108, no. 10: 1800–1809. 10.1017/s0007114512000128. 22370147 PMC3513714

[jpn70093-bib-0016] O'Byrne, S. M. , and W. S. Blaner . 2013. “Retinol and Retinyl Esters: Biochemistry and Physiology.” Journal of Lipid Research 54, no. 7: 1731–1743. 10.1194/jlr.r037648. 23625372 PMC3679378

[jpn70093-bib-0017] Pobisch, R. , and K. Onderscheka . 1976. “Die Vitamin‐A‐Hypervitaminose bei der Katze.” Wiener Tierärztliche Monatsschrift 63, no. 10: 283–294.

[jpn70093-bib-0018] Raila, J. , L. Brunnberg , F. J. Schweigert , and B. Kohn . 2010. “Influence of Kidney Function on Urinary Excretion of Albumin and Retinol‐Binding Protein in Dogs With Naturally Occurring Renal Disease.” American Journal of Veterinary Research 71, no. 11: 1387–1394. 10.2460/ajvr.71.11.1387. 21034333

[jpn70093-bib-0019] Raila, J. , I. Buchholz , H. Aupperle , G. Raila , H. A. Schoon , and F. J. Schweigert . 2000. “The Distribution of Vitamin A and Retinol‐Binding Protein in the Blood Plasma, Urine, Liver and Kidneys of Carnivores.” Veterinary Research 31, no. 6: 541–551. 10.1051/vetres:2000138. 11129798

[jpn70093-bib-0020] Raila, J. , S. Forterre , B. Kohn , L. Brunnberg , and F. J. Schweigert . 2003. “Effects of Chronic Renal Disease on the Transport of Vitamin A in Plasma and Urine of Dogs.” American Journal of Veterinary Research 64, no. 7: 874–879. 10.2460/ajvr.2003.64.874. 12856772

[jpn70093-bib-0021] Raila, J. , U. Neumann , and F. J. Schweigert . 2003. “Immunochemical Localization of Megalin, Retinol‐Binding Protein and Tamm‐Horsfall Glycoprotein in the Kidneys of Dogs.” Veterinary Research Communications 27, no. 2: 125–135. 10.1023/a:1022859120447. 12718506

[jpn70093-bib-0022] Raila, J. , R. Radon , A. Trüpschuch , and F. J. Schweigert . 2002. “Retinol and Retinyl Ester Responses in the Blood Plasma and Urine of Dogs After a Single Oral Dose of Vitamin A.” Journal of Nutrition 132, no. 6: 1673S–1675S. 10.1093/jn/132.6.1673s. 12042489

[jpn70093-bib-0023] Raila, J. , F. J. Schweigert , and B. Kohn . 2014. “Relationship Between Urinary Tamm‐Horsfall Protein Excretion and Renal Function in Dogs With Naturally Occurring Renal Disease.” Veterinary Clinical Pathology 43, no. 2: 261–265. 10.1111/vcp.12143. 24894070

[jpn70093-bib-0024] Raila, J. , T. E. Willnow , and F. J. Schweigert . 2005. “Megalin‐Mediated Reuptake of Retinol in the Kidneys of Mice Is Essential for Vitamin A Homeostasis.” Journal of Nutrition 135, no. 11: 2512–2516. 10.1093/jn/135.11.2512. 16251603

[jpn70093-bib-0025] Schweigert, F. , and V. Bok . 2000. “Vitamin A in Blood Plasma and Urine of Dogs Is Affected by the Dietary Level of Vitamin A.” International Journal for Vitamin and Nutrition Research 70, no. 3: 84–91. 10.1024/0300-9831.70.3.84. 10883401

[jpn70093-bib-0026] Schweigert, F. J. 1988. “Insensitivity of Dogs to the Effects of Nonspecific Bound Vitamin A in Plasma.” International Journal for Vitamin and Nutrition Research 58, no. 1: 23–25. 3384578

[jpn70093-bib-0027] Schweigert, F. J. , I. Buchholz , and K. Bonitz . 1998. “Effect of Age on the Levels of Retinol and Retinyl Esters in Blood Plasma, Liver and Kidney of Dogs.” International Journal for Vitamin and Nutrition Research 68, no. 4: 237–241. 9706498

[jpn70093-bib-0028] Schweigert, F. J. , J. Raila , and S. Haebel . 2002. “Vitamin A Excreted in the Urine of Canines Is Associated With a Tamm‐Horsfall Like Protein.” Veterinary Research 33, no. 3: 299–311. 10.1051/vetres:2002018. 12056481

[jpn70093-bib-0029] Schweigert, F. J. , E. Thomann , and H. Zucker . 1991. “Vitamin A in the Urine of Carnivores.” International Journal for Vitamin and Nutrition Research 61, no. 2: 110–113. 1917346

[jpn70093-bib-0030] Seawright, A. A. , P. B. English , and R. J. W. Gartner . 1967. “Hypervitaminosis A and Deforming Cervical Spondylosis of the Cat.” Journal of Comparative Pathology 77, no. 1: 29–IN6. 10.1016/s0021-9975(67)80004-5. 6030457

[jpn70093-bib-0031] Smets, P. M. Y. , E. Meyer , B. E. J. Maddens , L. Duchateau , and S. Daminet . 2010. “Urinary Markers in Healthy Young and Aged Dogs and Dogs With Chronic Kidney Disease.” Journal of Veterinary Internal Medicine 24: 65–72. 10.1111/j.1939-1676.2009.0426.x. 20041990

[jpn70093-bib-0032] Smith, F. R. , and D. S. Goodman . 1976. “Vitamin A Transport in Human Vitamin A Toxicity.” New England Journal of Medicine 294, no. 15: 805–808. 10.1056/nejm197604082941503. 943041

[jpn70093-bib-0033] Stephensen, C. , J. Alvarez , J. Kohatsu , R. Hardmeier , J. Kennedy, Jr. , and R. Gammon, Jr . 1994. “Vitamin A Is Excreted in the Urine During Acute Infection.” American Journal of Clinical Nutrition 60, no. 3: 388–392. 10.1093/ajcn/60.3.388. 8074070

[jpn70093-bib-0034] Sun, H. 2012. “Membrane Receptors and Transporters Involved in the Function and Transport of Vitamin A and Its Derivatives.” Biochimica et Biophysica Acta (BBA) ‐ Molecular and Cell Biology of Lipids 1821, no. 1: 99–112. 10.1016/j.bbalip.2011.06.010. 21704730 PMC3222080

[jpn70093-bib-0035] Wilson, D. E. , J. Hejazi , N. L. Elstad , C. Ing‐Fong , J. M. Gleeson , and P. H. Iverius . 1987. “Novel Aspects of Vitamin A Metabolism in the Dog: Distribution of Lipoprotein Retinyl Esters in Vitamin A‐Deprived and Cholesterol‐Fed Animals.” Biochimica et Biophysica Acta (BBA) ‐ Lipids and Lipid Metabolism 922, no. 3: 247–258. 10.1016/0005-2760(87)90047-6. 3689810

